# Viral Genomic Characterization and Replication Pattern of Human Polyomaviruses in Kidney Transplant Recipients

**DOI:** 10.3390/v12111280

**Published:** 2020-11-09

**Authors:** Lucia Signorini, Maria Dolci, Evaldo Favi, Caterina Colico, Mariano Ferraresso, Rosalia Ticozzi, Giuseppe Basile, Pasquale Ferrante, Serena Delbue

**Affiliations:** 1 Biomedical, Surgical and Dental Sciences, University of Milano, 20133 Milano, Italy; dolci@unimi.it (M.D.); rosalia.ticozzi@unimi.it (R.T.); pasquale.ferrante@unimi.it (P.F.); serena.delbue@unimi.it (S.D.); 2 Department of Clinical Sciences and Community Health, University of Milano, 20122 Milano, Italy; evaldofavi@gmail.com (E.F.); mariano.ferraresso@unimi.it (M.F.); 3 Kidney Transplantation, Fondazione IRCCS Ca’ Granda, Ospedale Maggiore Policlinico, 20122 Milano, Italy; caterina.colico@policlinico.mi.it; 4 Service of Legal Medicine, San Siro Clinical Institute, 20148 Milano, Italy; basiletraumaforense@gmail.com

**Keywords:** Human Polyomavirus JC (JCPyV), Human Polyomavirus BK (BKPyV), Merkel Cell Polyomavirus (MCPyV), kidney transplantation (KTx), urine, molecular characterization

## Abstract

Human Polyomavirus (HPyV) infections are common, ranging from 60% to 100%. In kidney transplant (KTx) recipients, HPyVs have been associated with allograft nephropathy, progressive multifocal leukoencephalopathy, and skin cancer. Whether such complications are caused by viral reactivation or primary infection transmitted by the donor remains debated. This study aimed to investigate the replication pattern and genomic characterization of BK Polyomavirus (BKPyV), JC Polyomavirus (JCPyV), and Merkel Cell Polyomavirus (MCPyV) infections in KTx. Urine samples from 57 KTx donor/recipient pairs were collected immediately before organ retrieval/transplant and periodically up to post-operative day 540. Specimens were tested for the presence of BKPyV, JCPyV, and MCPyV genome by virus-specific Real-Time PCR and molecularly characterized. HPyVs genome was detected in 49.1% of donors and 77.2% of recipients. Sequences analysis revealed the archetypal strain for JCPyV, TU and Dunlop strains for BKPyV, and IIa-2 strain for MCPyV. VP1 genotyping showed a high frequency for JCPyV genotype 1 and BKPyV genotype I. Our experience demonstrates that after KTx, HPyVs genome remains stable over time with no emergence of quasi-species. HPyVs strains isolated in donor/recipient pairs are mostly identical, suggesting that viruses detected in the recipient may be transmitted by the allograft.

## 1. Introduction

Human Polyomaviruses (HPyVs) are ubiquitous DNA viruses with a reported seroprevalence in the general population ranging from 60% to 100%, depending on the series [1,2]. After primary infection, usually contracted during childhood and asymptomatic, they often establish life-long latency in renal tubular epithelial cells and to a lesser degree in other cell types. In immunocompromised hosts, HPyVs can start uncontrolled replication and eventually lead to cell lysis, necrosis, and irreversible tissue damage [1]. 

Due to their chronic exposure to powerful immunosuppressive agents, kidney transplant (KTx) recipients have a greater risk of clinically significant HPyVs infections than healthy controls [3,4]. To date, the most relevant HPyVs detected in this particular group of patients are BK Polyomavirus (BKPyV), JC Polyomavirus (JCPyV), and Merkel Cell Polyomavirus (MCPyV) [2,4,5]. In particular, BKPyV has been linked to Polyomavirus-associated nephropathy (PVAN), JCPyV to progressive multifocal leukoencephalopathy (PML), and MCPyV to some forms of non-melanoma skin cancer [6]. Whether such complications are actually caused by reactivations of latent viruses of the recipient or rather by viruses transmitted by the donor through the allograft remains unclear [5].

HPyVs double-stranded DNA genome is circular and covalently closed. It is divided into an Early, a Late, and a Non-Coding Control Region (NCCR) containing the origin of DNA replication as well as the promoter/enhancer elements directing viral genes transcription [7]. The Early region, normally expressed prior to the initiation of viral DNA replication, encodes the Large and Small Tumour Antigens (LTAg and STAg) that play a key role in DNA replication, late viral genes transcription, and virions assembly [8]. The Late region encodes three viral capsid proteins (VP1, VP2, and VP3) required for capsid assembly and sustaining the uncoating process of the virus when it enters the host cells [9]. HPyV Late region could also contain open reading frames (*agnogene*), encoding for a 66 amino acid protein named agnoprotein—a regulatory cytoplasmic protein involved in viral release. The presence of the *agnogene* was demonstrated for JCPyV and BKPyV while there is no evidence that MCPyV expresses proteins analogous to agnoprotein [10,11,12]. Sequence variations analyses of the VP1 gene are routinely performed to discriminate between different viral genotypes [13]. Nevertheless, while BKPyV and JCPyV genotypes have been extensively characterized, MCPyV genotyping is still in progress [14,15,16,17,18,19,20,21,22,23,24]. The hypervariability of the NCCR, especially considering MCPyV, represents another topic of interest as differences in the sequences constituting this specific region are currently used to distinguish archetypal strains from rearranged ones and to obtain prognostic information regarding the course of the infection [13,25,26,27,28]. 

The present study aimed to evaluate origins, urinary replication patterns, and molecular characteristics of BKPyV, JCPyV, and MCPyV infections after KTx.

## 2. Materials and Methods 

### 2.1. Study Design 

In this single-center prospective observational study with an 18-month follow-up, we enrolled 57 consecutive KTx donor/recipient pairs. Both deceased and living donor transplants were considered. There were no exclusion criteria. Organ retrievals were carried out in hospitals under the area of influence of the Nord Italia Transplant (NITp) Organization whereas transplants were performed at the Fondazione IRCCS Ca’ Granda Ospedale Maggiore Policlinico (Milan) between June 2016 and December 2019. 

The main demographic and clinical characteristics of the participants are summarized in Table 1.

Baseline urine samples were collected immediately before organ retrieval (donors) and transplants (recipients). Follow-up specimens (recipients) were obtained according to the time-schedule reported in Table 2. 

The subjects involved signed a specific informed consent. The study was approved by the Fondazione IRCCS Ca’ Granda Ospedale Maggiore Policlinico Ethical Committee (protocol 2191) and conducted according to the World Health Organization Declaration of Helsinki and applicable regulatory requirements. 

### 2.2. DNA Isolation and Quantitative Real-Time PCR Assay

Urine samples from donors and recipients were collected in sterile containers and immediately transported to the laboratory where they were spun at 1500 rpm for 30′ and the supernatants were stored at −20 °C until use. Viral DNA was isolated from 150 µL of supernatant using the commercial kit NucleoSpin RNA virus kit (Macherey-Nagel GmbH & Co. KG, Dueren, Germany), according to manufacturer’s instructions. Quantitative Real-Time PCR (Q-RT-PCR) was performed using the Applied Biosystems 7500 Real-Time PCR System (Applied Biosystems, Foster City, CA, USA) with specific primers and TaqMan probe technology targeting the VP1 sequence of BKPyV and MCPyV or the LTAg sequence of JCPyV, adopting reaction conditions and thermal profiles previously described [29]. Strict precautionary measures were taken to avoid cross-contamination, including the use of separate rooms for nucleic acid extraction, preparation of amplification mixtures, execution of Q-RT-PCR, and nested PCR assays. Negative controls of DNA extraction and amplification were added to each run. Results were expressed as copies/mL of the urine sample.

### 2.3. HPyVs Molecular Characterization

HPyVs strains were molecularly characterized using different sets of specific nested PCR reactions. Amplification products were analyzed on 2% agarose gel in 0.5X TBE buffer. Positive PCR products were purified utilizing the QIAquick Gel Extraction Kit (QIAGEN, Hilden, Germany), following the manufacturer’s instructions. 

#### 2.3.1. Molecular Characterization of JCPyV

JCPyV genomes were molecularly characterized using nested PCR reactions for the amplification of the VP1 region and NCCR. Specific primer sets and thermal profiles were utilized for each nested PCR reaction (see Tables S1–S3 for details) [14,30,31,32,33,34,35,36]. The amplifications were carried out in a total volume of 50 µL containing 2 U of Taq DNA Polymerase (Solis BioDyne OÜ, Tartu, Estonia) in the presence of 10 µL of 5X Reaction Buffer supplied by the manufacturer and 3–10 µL of DNA template. For JCPyV VP1 external/internal amplification, the PCR reaction was finished adding 3 µL of 10 mM dNTPs solution, 5 µL of 25 mM MgCl_2_, and 3 µL of 10 µM primers. For JCPyV NCCR external/internal amplification, the PCR reaction was finished adding 2 µL of 10 mM dNTPs solution, 4 µL of 25 mM MgCl_2_, and 2 µL of 10 µM primers.

#### 2.3.2. Molecular Characterization of BKPyV

BKPyV genomes were molecularly characterized using nested PCR reactions for the amplification of the VP1 region and NCCR. Specific primer sets and thermal profiles were utilized for each nested PCR reaction (see Tables S1–S3 for details) [14,30,31,32,33,34,35,36]. The amplifications were carried out in a total volume of 50 µL containing 2 U of Taq DNA Polymerase (Solis BioDyne OÜ, Tartu, Estonia) in the presence of 10 µL of 5X Reaction Buffer supplied by the manufacturer and 3–10 µL of DNA template. For BKPyV VP1 external/internal amplification, the PCR reaction was finished adding 4 µL of 10 mM dNTPs solution, 4 µL of 25 mM MgCl_2_, and 2 µL of 10 µM primers. For BKPyV NCCR external/internal amplification, the PCR reaction was finished adding 2 µL of 10 mM dNTPs solution, 4 µL of 25 mM MgCl_2_, and 2,5/2 µL of 10 µM primers.

#### 2.3.3. Molecular Characterization of MCPyV

MCPyV genomes were characterized utilizing nested PCR reactions for the LTAg region and NCCR. Specific primer sets and thermal profiles were used for each nested PCR reaction (see Tables S1–S3 for details) [14,30,31,32,33,34,35,36]. The amplifications were carried out in a total volume of 50 µL containing 2 U of Taq DNA Polymerase (Solis BioDyne OÜ, Tartu, Estonia) in the presence of 10 µL of 5X Reaction Buffer supplied by the manufacturer and 3–10 µL of DNA template. For MCPyV VP1 external/internal amplification, the PCR reaction was finished adding 2 µL of 10 mM dNTPs solution, 2 µL of 25 mM MgCl_2_, and 2.5 µL of 10 µM primers. For MCPyV NCCR external/internal amplification, the PCR reaction was finished adding 2 µL of 10 mM dNTPs solution, 4/2 µL of 25 mM MgCl_2_, and 2 µL of 10 µM primers. Each reaction was completed with nuclease-free water up to a total volume of 50 µL. 

### 2.4. DNA Automatic Sequencing

DNA sequencing of the inner fragment of the nested PCR was carried out at an external facility (Eurofins Genomics GmbH, Ebersberg, Germany) using both forward and reverse primers. As previously described by Agostini et al. [37], for JCPyV genotyping we performed BLAST searches on the NCBI website (https://blast.ncbi.nlm.nih.gov) to assess sequences homology. As reported by Ault et al. [19] and Jensen et al. [38], BLAST searches were also performed to identify JCPyV NCCR rearrangements. BKPyV genotypes were determined following the classification method proposed by Jin et al. [39]. MCPyV sequences were aligned with those of the prototype strain MCC350 deposited in GenBank under the accession number EU375803 and analyzed according to the classification method proposed by Hashida et al. [35].

### 2.5. Immunosuppression and Concomitant Medications

As induction, low-immunological risk recipients (primary transplant, last panel-reactive antibody status < 50%, undetectable donor-specific antibody, and kidney retrieved from a living donor or a standard-criteria donation after brain death donor) were given intravenous basiliximab (Simulect^®^, Novartis, Basel, Switzerland) 20 mg on days 0 and day 4 whereas high-immunological risk patients received intravenous rATG (Thymoglobulin^®^, Genzyme, Cambridge, MA, USA) 5 mg/kg total-dose from day 0 to day 4. Pre- and post-transplant plasmapheresis along with a seven-day course of intravenous IgG up to 2 g/kg total-dose were selectively administered in case of donor-specific antibody > 2000 MFI at the time of transplant. Participants were also given intravenous methylprednisolone 500 mg on day 0, 250 mg on day 1, and 125 mg on day 2. As for maintenance, patients received oral tacrolimus (Adoport^®^, Novartis, Basel, Switzerland) 0.10 mg/kg/day and oral mycophenolate mofetil (Myfenax^®^, Teva, Petach Tikva, Israel) 2000 mg/day from day 0. Tacrolimus dose was adjusted to achieve a trough level of 8–12 ng/mL during the first post-transplant month and 6–8 ng/mL thereafter. From day 3, patients received oral prednisone 20 mg/day, progressively tapered to 5 mg/day after 1 month. For pneumocystis jirovecii and cytomegalovirus prophylaxis we used oral trimethoprim/sulfamethoxazole 80/400 mg/day three times per week for three months and oral valganciclovir (dose titrated according to renal function) for 3 months, respectively.

### 2.6. Statistical Analysis

Categorical and numerical variables were described using proportions, medians, and 1st–3rd interquartile ranges (IQR). Results were compared using Fisher’s exact test, Chi-square test or Mann–Whitney U test as appropriate. The significance of statistical tests was taken at a *p*-value < 0.05. Analyses were performed using SPSS (version 23.0; IBM Corp., Armonk, NY, USA). 

## 3. Results

### 3.1. HPyVs Viruria in the Study Cohort

As summarized in Table 3, HPyVs genomes were detected in the urine of 28/57 (49.1%) donors and 44/57 (77.2%) recipients (*p* = 0.0033). Among the donors with viruria, 25/28 (89.3%) tested positive for JCPyV, 3/28 (10.7%) for BKPyV, and 2/28 (7.1%) for MCPyV (*p* < 0.00001 comparing JCPyV vs. BKPyV and JCPyV vs. MCPyV). The proportions of viruric recipients positive for JCPyV, BKPyV, and MCPyV were 25/44 (56.8%), 21/44 (47.7%), and 17/44 (38.6%), respectively (*p* = ns). Notably, the differences observed in virus-specific prevalence between donors and recipients were all statistically significant (*p* = 0.0039 for JCPyV; *p* = 0.0017 for BKPyV, and *p* = 0.0029 for MCPyV). Coinfections were only recorded in the recipients’ group (19/44, 43.2% vs. 0/28, 0%; *p* < 0.00001). More in details, the following combinations were observed: JCPyV plus BKPyV (6/44, 13.6%), JCPyV plus MCPyV (8/44, 18.2%), BKPyV plus MCPyV (2/44, 4.5%), and JCPyV plus BKPyV plus MCPyV (3/44, 6.8%). Specific coinfection rates were not significantly different (*p* = ns). 

### 3.2. HPyVs Viruria in Urinary Samples

As summarized in Table 4, HPyVs genomes were detected in 272/551 (49.4%) urinary samples. More in details, 172/551 (31.2%) specimens tested positive for JCPyV, 62/551 (11.2%) for BKPyV, and 38/551 (6.9%) for MCPyV (*p* < 0.00001 comparing JCPyV vs. BKPyV and JCPyV vs. MCPyV; *p* = 0.016 comparing BKPyV vs. MCPyV). Coinfection was observed in 38/551 (6.9%) samples: JCPyV plus BKPyV in 10/551 (1.8%), JCPyV plus MCPyV in 24/551 (4.3%) and BKPyV plus MCPyV in 4/551 (0.7%).

### 3.3. HPyVs Urinary Load

**Figure 1:**
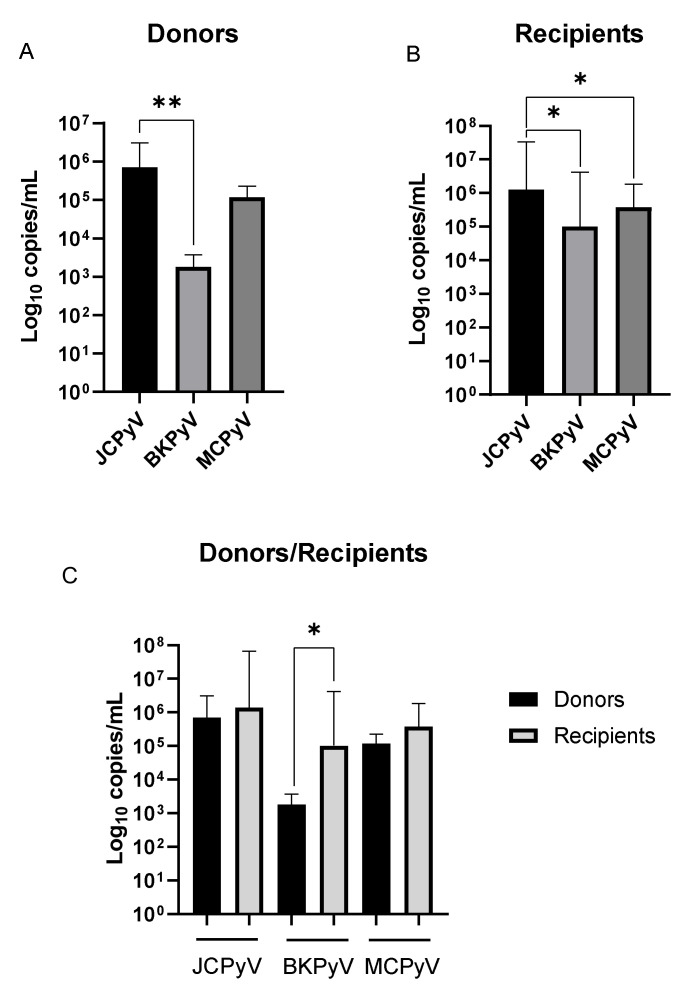
Median (IQR) virus-specific urinary loads in donors’ (**A**) and recipients’ (**B**) groups; direct comparison between donors’ and recipients’ median (IQR) virus-specific urinary loads (**C**). P values: ** *p* = 0.0043 (A), * *p* = 0.0166 (JCPyV vs. BKPyV and * *p* = 0.0158 JCPyV vs. MCPyV (B), * *p* = 0.0107 (C).

Median virus-specific urinary loads in the study cohort are reported in Table 5 and depicted in Figure 1. JCPyV showed the highest urinary load in both donors’ and recipients’ groups: 7.11 × 10^5^ copies/mL (IQR 9.51 × 10^4^ – 1.26 × 10^6^ copies/mL) and 1.29 × 10^6^ copies/mL (IQR 5.66 × 10^4^ − 3.17 × 10^7^), respectively (*p* = 0.0043 comparing JCPyV vs. BKPyV among donors; *p* = 0.0166 and *p* = 0.0158 comparing JCPyV vs. BKPyV and JCPyV vs. MCPyV among recipients. We also observed that BKPyV urinary load was significantly higher in the recipients’ group than donors’ one (1.02 × 10^5^ copies/mL, IQR 1.30 × 10^4^ − 3.69 × 10^6^ vs. 1.80 × 10^3^ copies/mL, IQR 1.18 × 10^3^ − 2.76 × 10^3^; *p* = 0.0107).

### 3.4. HPyVs Molecular Characterization

Donors’ and recipients’ urinary samples positive for HPyVs DNA as detected by Q-RT-PCR assay were amplified using HPyVs nested-PCR reactions and then sequenced for VP1 genes and NCCR to assess possible rearrangements. PCR reactions were performed on viral strains amplified in specimens collected from the donors at baseline and in specimens collected from the recipients on post-transplant day 1, 270, and 540. 

#### 3.4.1. JCPyV Molecular Characterization

JCPyV molecular characterization was carried out in 24 donors and 24 recipients. Sequence analysis of the NCCR demonstrated the presence of the archetypal strain, with several point mutations (data not shown), in all donors and recipients isolates, at every time points. As detailed in Table 6, VP1 gene sequence analysis in the donors’ group allowed to identify genotype 1a (6/24, 25%), genotype 1b (7/24, 29.2%), genotype 2a (1/24, 4.2%), genotype 2c (3/24, 12.5%), genotype 4 (5/24, 20.8%), and genotype 6 (1/24, 4.2%). Strains and genotypes distributions in the recipients’ group (Table 6) were concordant with those observed in the donor’s group with the only exception of 2/24 (8.3%) recipients who experienced a shift from genotype 1 to genotype 4 at 270 and 540 days after KTx. In both cases, genotype 4 was transmitted by the donor (Table 6 and Figure S1).

#### 3.4.2. BKPyV Molecular Characterization

BKPyV molecular characterization was performed in three donors and 13 recipients. Sequence analysis of the NCCR in the donors’ group demonstrated the presence of the archetypal strain (1/3, 33.3%) and the TU strain (1/3, 33.3%); due to technical issues, results were only available for 2 out of 3 donors. In the recipients’ group, we detected the WW strain (7/8, 87.5%) and the Dunlop strain (1/8; 12.5%). As described in Table 7 and depicted in Figure S2, VP1 gene sequence analysis among donors allowed to identify genotype Ia (1/3, 33.3%) and genotype Ib-1 (2/3, 66.6%). In the recipients’ group, genotypes were as follow: genotype Ia (1/13, 7.7%), genotype Ib-1 (8/13, 61.6%), and genotype Ib-2 (4/13, 30.8%). Matching donors and recipients, we found that strains and genotypes were concordant with the only exception of a single donor/recipient pair showing the WW strain genotype Ib-1 in the donor and the Dunlop strain genotype Ia in the recipient (Table 7 and Figure S2).

#### 3.4.3. MCPyV Molecular Characterization

MCPyV molecular characterization was carried out in two donors and 14 recipients. Sequence analysis of the NCCR region, performed in one donor and nine recipients, demonstrated the presence of the genotype IIa-2 in all subjects. VP1 gene sequence was analyzed in one donor and five recipients. With the exclusion of a few point mutations (data not shown), results demonstrated a complete alignment with the MCC350 strain. 

### 3.5. HPyV-Associated Diseases

No episodes of PML or MCPyV-related skin cancer were recorded. One patient with BKPyV viruria (and BKPyV viremia) developed PVAN during the follow-up. This recipient successfully managed to reduce the net state of immunosuppression. Following treatment, allograft function remained consistently stable over time with no evidence of rejection.

## 4. Discussion

KTx is universally considered the golden standard treatment of end-stage renal disease [3]. Due to a widespread application of aggressive immunosuppressive protocols, we have witnessed a remarkable reduction of acute rejection-related allograft losses over the years [40,41]. The drawback of such a great achievement is that we are now facing a significant rise in post-transplant infectious diseases and drug-related adverse effects [42,43,44]. Among infectious complications, those caused by HPyVs represent a strenuous challenge for the transplant community. In fact, the ubiquitous distribution of these specific viruses in the general population, their ability to establish asymptomatic latency, the severity of the conditions caused under immunosuppression, and the lack of proper antiviral therapies make it extremely difficult to develop adequate prevention and treatment strategies [1,45,46]. In this context, a better understanding of HPyVs epidemiology, biology, and behavior in the immunocompromised host is paramount.

The main objectives of the present study were to evaluate the prevalence of JCPyV, BKPyV, and MCPyV in the actual KTx donors and recipients pool, to investigate whether episodes of viral replication during the various post-transplant phases are due to reactivations of previously acquired latent viruses or rather primary infections transmitted by the allograft, to assess possible interactions between different HPyVs, and to further characterize clinically relevant strains and genotypes. 

Testing urinary samples collected from an Italian pool of KTx donor/recipient pairs, we found that the overall prevalence of HPyVs was about 50% among donors and 80% among recipients. Finding a higher percentage of positive recipients compared to donors might represent an ideal situation, since recipients would be more likely to develop a prompt immunological response against the HPyVs, in case of reactivation. 

These results are in line with those from other reports demonstrating active replication of HPyVs in up to 60% of healthy individuals [2,47,48,49,50,51,52] and 75% of adult transplanted patients [53]. They also confirm the key role of immunosuppression in promoting HPyVs replication as the proportion of recipients with viruria was significantly higher than donors and progressively increased along with the duration of the exposure to immunosuppressants [2,47,48,49,50,51,52]. 

In this series, JCPyV was the most frequently detected HPyV in both donors’ and recipients’ groups. High JCPyV replication rates in immunocompetent subjects have been already demonstrated [2,47,48,49,50,51,52]. On the contrary, available data on KTx recipients remain conflicting [53,54]. Even though some authors have shown that JCPyV urinary prevalence can be as high as 75% and greater than BKPyV [53,54], the majority of reports suggest that BKPyV viruria is actually more frequent than JCPyV [55,56]. A possible reason behind this discrepancy is that most of the previously published studies evaluating BKPyV prevalence in urine samples focused on patients with documented viremia, symptomatic infection, or biopsy-proven PVAN and therefore more likely to have concomitant BKPyV viruria compared to our population of prospectively followed-up asymptomatic KTx recipients [55,56]. The fact that the cumulative incidence of clinically significant BKPyV infection observed during the present study was on the lower end of the values reported in the literature may also have contributed. Results obtained assessing MCPyV prevalence are more difficult to interpret as there is still a generalized lack of information regarding this specific virus in the KTx setting. Overall, they seem aligned with the few reports available showing relatively low percentages of viruric individuals among both donors and recipients [51,55,57]. 

In the present study, the relationship between immunosuppressive medications and HPyVs prevalence or replication pattern in urine was not investigated. In previous research, we observed that high-immunological risk characteristics such as Afro-Caribbean ethnicity, donor-recipient HLA mismatch > 4, and panel-reactive antibody test > 50% were predictors of BKPyV infection and viremia after KTx but no statistically significant associations between specific induction agents or maintenance drugs could be detected [58]. In-depth analysis of the effects of different anti-rejection treatments on HPyV infections susceptibility as well as targeted investigations aiming to determine a possible dose-effect or concentration-effect of tacrolimus and mycophenolate mofetil on HPyVs replication in blood and urine are certainly warranted.

Quantitative analysis of HPyVs viruria after transplantation demonstrated that JCPyV exhibited the highest viral load, followed by MCPyV and BKPyV. As previously reported by our group and others authors, these findings confirm high JCPyV urinary loads in patients receiving immunosuppressive therapy [51,59,60] and further support the hypothesis that, similarly to BKPyV and JCPyV, MCPyV may persist in renal tubular epithelial cells and eventually reactivate in case of transient alteration of the net state of immunosuppression [51,55].

JCPyV NCCR sequence analysis demonstrated that all tested subjects were infected by the archetypal strain. Data on JCPyV NCCR molecular characterization in KTx patients are extremely scarce. Therefore, direct comparison with other reports remains difficult if not impossible. In a similar study by Yin et al. [60], multiple JCPyV strains (namely, CY, TW1, and TW3) were detected with numerous episodes of simultaneous replication whereas strains with modified NCCR archetypal structure were identified by Seppälä and colleagues [59] performing a full-length JCPyV strain analysis on both plasma and urine samples. 

Using JCPyV VP1 gene sequence analysis, we found that the most frequently represented genotypes were 1a and 1b, followed by genotypes 2, 4, and 6. As genotypes 1a and 1b represent the predominant genotypes in Europe [14,22], this result suggests that JCPyV genotypes distribution in KTx recipients may simply resemble JCPyV genotypes geographical distribution [53,54,59,61,62]. Importantly, a very strong concordance between donor and recipient genotypes was observed thus confirming the hypothesis that post-transplant JCPyV replication is mostly due to de novo acquired infections transmitted by the donor through the allograft [61,63]. 

BKPyV NCCR and VP1 sequences analysis showed that only half of our donor/recipient pairs with viruria actually shared the same viral strain and genotype. Considering the limited amount of BKPyV infections recorded in this series, no firm conclusions can be drawn. However, we can reasonably speculate that at least some episodes of BKPyV replication occurring during the post-transplant course may be due to latent viruses previously acquired by the recipient and reactivated under immunosuppression. 

Interestingly, in one donor and one recipient, we detected rearranged forms of BKPyV NCCR already described in the literature and often linked to BKPyV-associated diseases [34,64]. Other studies assessing BKPyV NCCR genetic modifications in KTx recipients showed the presence of the archetypal and AS strains in renal allograft biopsies [55,56] and demonstrated BKPyV strains with few single-nucleotide substitutions within P, Q, R, and S blocks in blood and urine samples [34,65,66]. However, the clinical relevance of specific BKPyV NCCR rearrangements is still unclear and larger populations, as well as the longer follow-up, are needed to further address this topic. 

In our experience, genotype 1b was the most commonly detected BKPyV genotype. This finding is overall in line with current evidence demonstrating the predominance of genotype 1 across the world. Nevertheless, considering the specific country of origin of the vast majority of the patients enrolled in the present study (Italy), it does not match the geographical distribution reported in the general population [52,62,67]. 

Finally, our sequences analysis of MCPyV amplicons showed the presence of the IIa-2 strain, already isolated from the skin of healthy individuals and patients with malignancy in several countries all around the world [35]. To date, there is only one report on MCPyV NCCR molecular characterization. According to it, there is a relationship between MCPyV genotypes and ethnicity [54].

## 5. Conclusions

This prospective observational study with an 18-month follow-up investigated the prevalence, viral load, and molecular characteristics of JCPyV, BKPyV, and MCPyV in urine samples collected from a cohort of KTx donor/recipient pairs. Our data show that: (a) HPyVs genome remains stable after transplantation with no de novo generation of quasispecies; (b) the vast majority of HPyVs strains isolated in donor/recipient pairs are identical thus suggesting that episodes of viral replication detected after transplantation may be more likely due to viruses transmitted by the donor through the allograft rather than reactivated viruses previously acquired by the recipient. 

## Figures and Tables

**Table 1:** Demographic and clinical characteristics of kidney transplant donors and recipients.

	Donors (n = 57)	Recipients (n = 57)
Variables	Mean ± SD or n (%)	
Male:female	26:24 *	32:25
Country of origin ≠ Italy	4/53 ^##^ (7.5)	7/50 ^###^ (14)
Age (years)	55.2 ± 13.2 **	44.3 ± 17.9 ***
Living:deceased	23:29 ^#^	-
Basiliximab	-	31/52 ^$^ (59.6)
Tacrolimus	-	51/52 ^$^ (98.1)
Mycophenolate mofetil	-	50/52 ^$^ (96.1)

* 7 (12.3%) of 57 missing cases; ** 8 (14%) of 57 missing cases; *** 1 (1.8%) of 57 missing cases; ^#^ 5 (8.8%) of 57 missing cases; ^##^ Foreign countries: Albania, Sri Lanka, and Colombia; 1 (25%) of 4 missing case; ^###^ Foreign countries: Kenya, Ethiopia, Tunisia, Austria, Lebanon; 2 (28.6%) of 7 missing cases; ^$^ 5 (8.8%) of 57 missing cases.

**Table 2:** Urinary specimen’s collection time points.

Days After Transplantation	0	1	15	30	60	90	180	270	360	540
Code	T0	T1	T2	T3	T4	T5	T6	T7	T8	T9

**Table 3:** Human Polyomavirus (HPyV) prevalence in kidney transplant donors and recipients.

	JCPyV +/tot (%)	BKPyV +/tot (%)	MCPyV +/tot (%)	HPyV/tot (%)
Donors *	25/28 (89.3) ^#,$,&^	3/28 (10.7) ^^,$^	2/28 (7.1) °^,&^	28/57 (49.1) ^+^
Recipients **	25/44 (56.8) ^#^	21/44 (47.7) ^^^	17/44 (38.6) °	44/57 (77.2) ^+^
Total	50 ^α^/72 (69.4) ^§,£^	24 ^β^/72 (33.3) ^§^	19 ^π^/72 (26.4) ^£^	72/114 (63.1)

* Two coinfection, ** Nineteen coinfection, ^α^ Nineteen donor/recipient pairs, ^β^ Two donor/recipient pair, ^π^ One donor/recipient pair, ^#,^,°,+,$,&,§,£^ *p* < 0.05.

**Table 4:** HPyVs prevalence in urinary samples collected from kidney transplant donors and recipients.

JCPyV +/tot (%)	BKPyV +/tot (%)	MCPyV +/tot (%)	HPyV/tot (%)
172/551 (31.2) *^,°,#^	62/551 (11.2) *^,^,#,$^	38/551 (6.9) ^°,^,#,$^	272/551 (49.4)

* Ten JCPyV/BKPyV coinfections, ° Twenty-four JCPyV/MCPyV coinfections, ^^^ Four BKPyV/MCPyV coinfections. ^#^ *p* < 0.0001, ^$^ *p* < 0.05.

**Table 5:** HPyVs urinary loads in kidney transplant donors and recipients.

	Median Viral Load Copies/mL (IQR *)			
	JCPyV	BKPyV	MCPyV	HPyVs
Donors	7.11 × 10^5 °^ (1.17 × 10^6^)	1.80 × 10^3 °,$^ (1.58 × 10^3^)	1.18 × 10^5^ (1.10 × 10^5^)	3.64 × 10^5^ (1.15 × 10^6^)
Recipients	1.29 × 10^6 #,&^ (3.16 × 10^7^)	1.02 × 10^5 #,$^ (3.67 × 10^6^)	3.76 × 10^5 &^ (1.47 × 10^6^)	5.65 × 10^5^ (1.54 × 10^7^)
Total	1.01 × 10^6^ (2.57 × 10^7^)	6.39 × 10^4^ (2.60 × 10^6^)	3.67 × 10^5^ (1.34 × 10^6^)	5.42 × 10^5^ (1.16 × 10^7^)

* Interquartile range = 75° percentile—25° percentile; ^°,#,&,$^ *p* < 0.05.

**Table 6:** JC Polyomavirus (JCPyV) genotypes distribution in urine samples from kidney transplant donors and recipients.

JCPyV Genotype	Positive Donors (n = 24) (%)	Positive Recipients (n = 24) (%)
1a	6 (25.0%)	7* (25.0%)
1b	7 (29.2%)	7 (29.2%)
2a	1 (4.2%)	1 (4.2%)
2c	3 (12.5%)	2 (8.3%)
4	5 (20.8%)	6 * (25.0%)
6	1 (4.2%)	1 (4.2%)
Without identification	1 (4.2%)	-

* T1 genotype, switched to genotype 4 in the following time points in two recipients. *n* = number of donors/recipients.

**Table 7:** BK Polyomavirus (BKPyV) genotypes distribution in urine samples from kidney transplant donors and recipients.

BKPyV Genotype	Positive Donors (n = 3) (%)	Positive Recipients (n = 13) (%)
Ia	1 (33.3%)	1 (7.7%)
Ib-1	2 (66.7%)	8 (61.5%)
Ib-2	-	4 (30.8%)

## Supplementary Materials

The following are available online at https://www.mdpi.com/1999-4915/12/11/1280/s1, Table S1: Primer sequences for HPyVs nested PCR, Table S2: Thermal profiles for NCCR HPyVs nested PCR, Table S3: Thermal profiles for VP1 HPyVs nested PCR, Figure S1: Sequence of the JCPyV Viral protein 1 (VP1) gene amplified from urine samples (nucleotide 1710-1924; ACCESSION NC_001699), Figure S2: Sequence of the BKPyV Viral protein 1 (VP1) gene amplified from urine samples (nucleotide 1681-1958; ACCESSION V01108 J02038).
