# Supplementary material for: Viral Genomic Characterization and Replication Pattern of Human Polyomaviruses in Kidney Transplant Recipients
Source: Viruses. 2020 Nov 9;12(11):1280. doi: 10.3390/v12111280 (PMC7696855; doi:10.3390/v12111280)
Supplement: Supplementary file 1 [file viruses-12-01280-s001.pdf]

Table S1. Primer sequences for HPyVs nested PCR

	Region	Primers	Position	Sequence	Fragment length
JCV[*]	NCCR[Δ]	JRE-1	4989-5009	5′-CCTCCCTATTCAGCACTTTGT-3′	678 bp
		LP-2	518-537	5′-TGCGGCACCCATGAACCTGA-3′	
		RFOR	5085-5104	5′-GCCTCCACGCCCTTACTACT-3′	353 bp
		RREV	291-310	5′-CAGAAGCCTTACGTGACAGC-3′	
	VP1[β]	VP1-1	1569-1592	5′-TTGACTCAATTACAGAGGTAGAAT-3′	796 bp
		VP1-4a	2330-2353	5′-AGAAATTGGGTAGGGGTTTTTAAC-3′	
		VP1-1	1569-1592	5′-TTGACTCAATTACAGAGGTAGAAT-3′	357 bp
		JLP-16	1902-1924	5′-TAAAGCCTCCCCCCCAACAGAAA-3′	
BKV[°]	NCCR[π]	BKTT1	5106-5134	5′-AAGGTCCATGAGCTCCATGGATTCTTCC-3′	694 bp
		BKTT2	630-657	5′-CTAGGCCCCCAAAAGTGCTAGAGCAGC-3′	
		BR-P1	82-101	5′-TTGAGAGAAAGGGTGGAGGC-3′	276 bp
		BR-P2	339-358	5′-GCCAAGATTCCTAGGCTCGC-3′	
	VP1[ϒ]	VP1-7	1480-1500	5′-ATCAAAGAACTGCTCCTCAAT-3′	537 bp
		VP1-2R	2038-2059	5′-GCACTCCCTGCATTTCCAAGGG-3′	
		327-1	1630-1649	5′-CAAGTGCCAAAACTACTAAT-3′	327 bp
		327-2	1956-1937	5′-TGCATGAAGGTTAAGCATGC-3′	
MCV[#]	NCCR[Σ]	ORIF1	4832-4853	5′-AGAGAGCCTATACCACTAACAG-3′	502 bp
		ORIR1	5314-5334	5′-ACATGATTGAACTTTTATTGC-3′	
		ORIF2	5077-5100	5′-AATTTCACCAATATTGGCCAGCAG-3′	203 bp
		ORIR2	5261-5280	5′-GAGGCGGAGTTTGACTGAT-3′	
	VP1[α]	VP1 FwE	3174-3193	5′-GGCTTTCTTTTTGAGAGGCCT-3′	440 bp
		VP1 RevE	3613-3592	5′-TCCTTTGCATAGAGGGCCCACT -3′	
		VP1 FwI	3276-3297	5′-TTGGGTAAACAGTTTTCTCCTG-3′	240 bp
		VP1 RevI	3515-3493	5′-AGTAACATTAAAATATCTAGGCA-3′	

[*]Reference Mad1 strain
[°]Reference Dunlop strain
[#]Reference strain EU375803
[Δ]Reference [30]
[β]Reference [14,31]
[π]Reference [32,33]
[ϒ]Reference [34]
[Σ]Reference [35]
[α]Reference [36]

Table S2. Thermal profiles for NCCR HPyVs nested PCR

	Denaturation (time)	Annealing (time)	Extension (time)	Number of cycles
		**Outer/Inner reaction**		
		95°C for 5′		
**JCPyV**		59°C/63°C (30″)		35
**BKPyV**	95°C (30″)	60°C/63°C (30″)	72°C (30″)	30
**MCPyV**		54°C/58°C (30″)		35/30
		72°C for 5′		

Table S3. Thermal profiles for VP1 HPyVs nested PCR

	Denaturation (time)	Annealing (time)	Extension (time)	Number of cycles
		**Outer/Inner reaction**		
		95°C for 5′		
**JCPyV**		63°C/66°C (30″)		30
**BKPyV**	95°C (30″)	60°C/58°C (30″)	72°C (30″)	30
**MCPyV**		58°C/54°C (30″)		30
		72°C for 5′		

Figure S1. Sequence of the JCPyV Viral protein 1 (VP1) gene amplified from urine samples (nucleotide 1710-1924; ACCESSION NC_001699)

JCPyV

```
                                                                   (1771)         (1786)(1790)
Type 1a  acagtgtggccagaattccactacccaatctaaatgaggatctaacctgtggaaatatactcatgtgggaggctgtgaccttaaaaactgagg
Type 1b  acagtgtggccagaattccactacccaatctaaatgaggatctaacctgtggaaatatactcatgtgggaggctgtgaccttaaaaactgagg
Type 2a  acagtgtggccagaattccactacccaatctaaatgaggatctaacctgtggaaatatactAatgtgggaggctgtKaccttaaaaactgagg
Type 2c  acagtgtggccagaattccactacccaatctaaatgaggatctaacctgtggaaatatactAatgtgggaggctgtTaccttaaaaactgagg
Type 4   acagtgtggccagaattccactacccaatctaaatgaggatctaacctgtggaaatatactcatgtgggaggctgtgaccttaaaaactgagg
Type 6   acagtgtggccagaattccactacccaatctaaatgaggatctaacctgtggaaatatactAatgtgggaggctgtgaccCtaaaaactgagg

                 (1813) (1818)                    (1843)  (1850)           (1869-1870)
Type 1a  ttataggggtgacaagtttgatgaatgtgcactctaatgggcaagcaactcatgacaatggtgcagggaagccagtgcagggcaccagctttc
Type 1b  ttataggggtgacaagtttgatgaatgtgcactctaatggTcaagcaGctcatgacaatggtgcagggaagccagtgcagggcaccagctttc
Type 2a  ttataggggtRacaaCtttgatgaatgtgcactctaatggTcaagcaactcatgacaatggtgcaggAaagccagtgcagggcaccagctttc
Type 2c  ttataggggtgacaaCtttgatgaatgtgcactctaatggTcaagcaGctcatgacaatggtgcaggAaagccagtgcagggcaccagctttc
Type 4   ttataggggtgacaaCtttgatgaatgtgcactctaatgggcaagcaactcatgacaatggtgcagCAaagccagtgcagggcaccagctttc
Type 6   ttataggggtgacaaCtttgatgaatgtgcactctaatggTcaagcaactcatgacaatggtgcagggaagccagtgcagggcaccagctttc

Type 1a  attttttttctgttgggggggaggcttta
Type 1b  attttttttctgttgggggggaggcttta
Type 2a  attttttttctgttgggggggaggcttta
Type 2c  attttttttctgttgggggggaggcttta
Type 4   attttttttctgttgggggggaggcttta
Type 6   attttttttctgttgggggggaggcttta
```

K=G or T; R=A or G

Figure S2. Sequence of the BKPyV Viral protein 1 (VP1) gene amplified from urine samples (nucleotide 1681-1958; ACCESSION V01108 J02038)

BKPyV

(1687) (1698)

Type Ia actggg**G**tagatgctat**T**acagaggtagaatgcttcctaaacccagaaatgggggatccagatgaaaaccttaggggctttagtctaaagcta

Type Ib-1 actggg**G**tagatgctat**A**acagaggtagaatgcttcctaaacccagaaatgggggatccagatgaaaaccttaggggctttagtctaaagcta

Type Ib-2 actggg**C**tagatgctat**A**acagaggtagaatgcttcctaaacccagaaatgggggatccagatgaaaaccttaggggctttagtctaaagcta

(1809) (1860)

Type Ia agtgctgaaaatgactttagcagtgatagcccaga**G**agaaaaatgcttccctgttacagcacagcaagaattcccctccccaattt**A**aatgag

Type Ib-1 agtgctgaaaatgactttagcagtgatagcccaga**A**agaaaaatgcttccctgttacagcacagcaagaattcccctccccaattt**A**aatgag

Type Ib-2 agtgctgaaaatgactttagcagtgatagcccaga**M**agaaaaatgcttccctgttacagcacagcaagaattcccctccccaattt**A**aatgag

(1887) (1908) (1923)

Type Ia gacctaacctgtggaaatct**A**ctgatgtgggaggctgtaac**T**gtacaaacagaggt**T**attggaataactagcatgcttaaccttcatgcagg

Type Ib-1 gacctaacctgtggaaatct**A**ctgatgtgggaggctgtaac**T**gtacaaacagaggt**C**attggaataactagcatgcttaaccttcatgcagg

Type Ib-2 gacctaacctgtggaaatct**A**ctgatgtgggaggctgtaac**A**gtacaaacagaggt**C**attggaataactagcatgcttaaccttcatgcagg

M=A or
